# Left Behind After Birth: A Cross‐Sectional Analysis of Factors Associated With Postnatal Care Use in Sub‐Saharan Africa

**DOI:** 10.1155/jp/3035162

**Published:** 2026-04-24

**Authors:** Alex Bawuah, Michael Sarfo, Linus Baatiema, Francis Appiah, Sanni Yaya

**Affiliations:** ^1^ School of Global Studies, Faculty of Social Science, University of Sussex, Brighton, UK, sussex.ac.uk; ^2^ School of Human and Health Sciences, University of Huddersfield, Huddersfield, UK, hud.ac.uk; ^3^ Ghana Health Service, Upper West Regional Health Directorate, Wa, Ghana, ghanahealthservice.org; ^4^ Centre for Migration, Security and International Relations, University of Business and Integrated Development Studies, Wa, Ghana; ^5^ School of Graduate Studies, Lingnan University, Tuen Mun, Hong Kong, China, ln.edu.hk; ^6^ Department of Social Sciences, Berekum College of Education, Berekum, Bono Region, Ghana, becoled.edu.gh; ^7^ Department of Population, Family and Reproductive Health, Kwame Nkrumah University of Science and Technology, Kumasi, Ghana, knust.edu.gh; ^8^ The George Institute for Global Health, Imperial College London, London, UK, imperial.ac.uk

**Keywords:** antenatal care, child health, DHS, health equity, maternal health, postnatal care, sub-Saharan Africa

## Abstract

**Background:**

Global commitment towards improving maternal and newborn health outcomes is pronounced, yet this is yielding little results as gaps in postnatal care (PNC) coverage persist across sub‐Saharan Africa (SSA). Low uptake of PNC partly contributes to preventable maternal and neonatal morbidity and mortality within the early days after birth. A better understanding of the individual, systemic and contextual factors influencing PNC uptake is vital for shaping effective policies and targeted interventions. This study explores the prevalence and factors associated with PNC utilisation among women and newborns across 27 SSA countries, aiming to identify key factors influencing service uptake to inform targeted interventions.

**Methods:**

We used data from the Demographic and Health Surveys (2015–2024), including 136,721 women aged 15–49 who had a live birth within 2 years of the survey. Andersen′s behavioural model guided the selection of predisposing, enabling and need factors. Multivariable logistic regression was employed to identify determinants of PNC utilisation for both mothers and newborns, adjusting for sampling design.

**Results:**

The overall prevalence of PNC utilisation was 75.24% for mothers and 79.37% for children. PNC coverage varied significantly across countries, with the highest maternal utilisation in Ghana (94.9%) and the lowest in Malawi (47.5%). For newborns, coverage ranged from 96.7% in South Africa to 43.1% in Ethiopia. Significant factors associated with PNC uptake included caesarean delivery (AOR = 3.64; 95% CI: 3.33–3.98), four or more antenatal visits (AOR = 1.24; 95% CI: 1.20–1.29), higher maternal education (AOR = 1.32; 95% CI: 1.17–1.49), health insurance, employment status and frequent media exposure. Conversely, women with unintended pregnancies and limited access to health information were less likely to utilise PNC services.

**Conclusion:**

PNC utilisation in SSA remains suboptimal and uneven. Addressing barriers related to education, healthcare access, media exposure and financial protection is essential to improving postnatal outcomes. Targeted, context‐specific strategies are needed to accelerate progress towards achieving Sustainable Development Goal 3 in the region.

## 1. Introduction

Postnatal care (PNC) refers to a critical period for mothers, babies, spouses, fathers, caregivers and family members, encompassing all essential care and support provided to the mother and baby following childbirth, continuing up to 42 days after delivery [1–3]. According to the World Health Organization (WHO), in 2023 alone, approximately 700 women died each day from preventable causes related to pregnancy and childbirth, amounting to a total of 260,000 maternal deaths during and following pregnancy and childbirth [4]. Of these, about 92% occurred in low‐ and lower–middle‐income countries, with sub‐Saharan Africa and Southern Asia accounting for the highest burden, representing 87% (225,000) of maternal deaths, and sub‐Saharan Africa alone contributing 70% (182,000) of the total mortality figures. Most of these maternal deaths occur in the postnatal period, with postpartum haemorrhage (PPH) being the leading cause [5, 6].

The care provided to mothers and newborns during the first 6 weeks after childbirth includes physical examinations, mental health assessments, health education, guidance on breastfeeding, contraceptive counselling, neonatal care, home visits and follow‐up for any pregnancy‐induced or pre‐existing medical conditions [7, 8]. Despite the well‐documented benefits of PNC for both mothers and their newborns [9, 10], previous studies have highlighted a range of socioeconomic and cultural factors that influence the utilisation of PNC services [11–14]. For example, a study conducted in Nigeria by Olajubu et al. [15] reported that although a significant proportion of respondents (98.8%) demonstrated good knowledge of PNC, only 22% actually utilised these services. The study further identified negative attitudes of healthcare workers, employment status, level of education and financial constraints as key barriers to service utilisation. Similarly, research conducted in Ghana indicated that a lack of maternal education regarding recommended practices and institutional protocols, as well as communication barriers, prevented many women from accessing PNC services [16].

According to a 2019 UNICEF report, approximately 60% of mothers and 43% of newborns globally received a postnatal health check within the recommended timeframeA [17]. In many African countries, a considerable proportion of women and newborns do not return to health facilities after childbirth, suggesting that PNC remains one of the most neglected components of reproductive and child health services. Utilisation rates remain low and vary significantly across sub‐Saharan Africa, with reported coverage of 47% in Kenya, 41.2% in Nigeria, 43.53% in Tanzania, 43.55% in Zambia, 51% in Uganda and 57.5% in Ethiopia [18–21].

In view of global efforts to reduce maternal and child mortality and to address postpartum health challenges, particularly within low‐ and middle‐income countries, this study adopts the Andersen′s Behavioural Model Of Health Services Use [22, 23], which has been widely applied in health services research to assess factors associated with immediate PNC among our study population. The model posits that health service utilisation is shaped by three broad categories of factors. These include predisposing factors such as age, education, parity and exposure to mass media; enabling factors such as household wealth, health insurance coverage, place of residence and access to healthcare facilities; and need factors such as the number of antenatal care (ANC) visits, mode of delivery (for example caesarean section) and whether the pregnancy was wanted or unintended. In this study, PNC is defined as the immediate health check received before discharge from the health facility, based on the Demographic and Health Survey (DHS) measure. This captures only facility‐based immediate PNC and does not include follow‐up visits within the first 6 weeks postpartum; therefore, findings should not be interpreted as reflecting utilisation of the full PNC continuum.

Against this backdrop, this study is aimed at examining the factors associated with PNC utilisation among women and newborns, and to identify key gaps and barriers in the delivery of postnatal services in the sub‐Saharan African context, with the goal of contributing to the achievement of Sustainable Development Goal 3 by 2030. Specifically, this study offers an original contribution to the discourse on PNC among women in Africa by drawing on the most recent data from 27 countries (2015–2024) to examine factors associated with immediate PNC among women and newborns in sub‐Saharan Africa for both women and newborns, using Andersen′s model as a guiding framework.

## 2. Methods

### 2.1. Data Source

This study drew on the most recent DHS data from 27 sub‐Saharan African countries conducted between 2015 and 2024. The countries included Nigeria, Ethiopia, Tanzania, Uganda, South Africa, Ghana, Senegal, Zambia, Zimbabwe, Mozambique, Lesotho, Rwanda, Sierra Leone, Liberia, Guinea, Benin, Burkina Faso, Mali, Mauritania, Gabon, Madagascar, Côte d’Ivoire, Burundi, Angola, Cameroon, Gambia and Malawi. The DHS programme provides nationally representative data across more than 90 low‐ and middle‐income countries, using standardised data collection procedures to ensure comparability between settings [24]. Information is collected using structured questionnaires covering key health indicators such as mortality, family planning, morbidity, fertility, maternal and child health [25]. A stratified two‐stage sampling design is employed to collect data. In the first stage, enumeration areas are selected from national sampling frames, followed by the selection of households within each cluster in the second stage. Further details on DHS sampling procedures and data collection methods can be found in the work of Aliaga and Ren [26].

Data for this study were obtained from the DHS women′s recode (IR) files. The pooled sample comprised 408,431 women within the reproductive age range of 15–49 years. For this study, the sample is limited to women of reproductive age (15–49) who had a live birth in the 2 years preceding the survey and complete information on all study variables. Focusing on recent births reduces recall bias for the timing of postnatal checks and ensures relevance to current health system performance. Approximately 65% (265,453) of respondents in the pooled DHS dataset had missing information on outcome variables—PNC for the mother and for the child. The missingness largely reflects women who did not have a live birth in the 2 years preceding the survey or for whom PNC information was not collected. A higher proportion of the women with missing data had no children, were younger, resided in rural areas, had limited media exposure, had less than four ANC visits, had no health insurance, were in the richest wealth group and had secondary education. Detailed characteristics of excluded observations are provided in Table S1. After excluding missing observations, the final analytic sample comprised 136,721 women. Missing data were handled using listwise deletion to ensure consistency across variables included in multivariable analyses.

### 2.2. Variables

The study outcome variable is PNC utilisation. This was assessed using two variables: PNC for the mother and PNC for the child.

PNC for the mother was assessed using the DHS variable “m62_1”, which records whether the respondent received a health check after delivery before discharge. This variable is coded as “1” if the respondent received the health check and “0” if they did not.

PNC for the baby was assessed using the DHS variable “m74_1”, which records whether the child received a health check after delivery before discharge. This variable is coded as “1” if the child received the health check and “0” if they did not.

The analysis included several explanatory variables informed by prior literature and data availability within the DHS [27, 28]. These comprised mode of delivery (caesarean section: yes, no), parity (1–2, 3–5, ≥ 6) and number of ANC visits (< 4, ≥ 4). Sociodemographic characteristics considered include maternal age (15–19, 20–24, 25–29, 30–34, 35–39, 40–44 and 45–49), level of education (no education, primary, secondary and higher), household wealth index (poorest, poorer, middle, richer and richest), place of residence (urban and rural) and employment status (employed and unemployed). Access‐related factors included health insurance coverage (insured and uninsured) and perceived difficulty in accessing healthcare due to distance (yes, no). Reproductive intention was captured using pregnancy wantedness (then, later and no more). Exposure to mass media was assessed through frequency of reading newspapers or magazines, watching television and listening to the radio (not at all, less than once a week, at least once a week and almost every day).

### 2.3. Data Analysis

The analysis was conducted using Stata Version 18. Summary statistics were generated to characterise the study sample and to estimate the prevalence of PNC utilisation for both mother and child across the 27 SSA countries. Given the binary nature of the outcome variables, a binary multivariable logistic regression model is employed to assess the factors associated with PNC utilisation [28, 29]. The complex survey design of the DHS was accounted for in all regression analyses (by incorporating sampling weights, stratification and primary sampling units) using the “svy” command, in line with DHS analytical guidelines [30].

### 2.4. Ethical Consideration

The study was based on secondary data from the DHS Program. Permission to use the data was obtained by submitting a formal request through the DHS website (https://dhsprogram.com/). The datasets are anonymised and made accessible to researchers following approval. The data are deidentified and were originally collected with informed consent; hence, this study did not require additional ethical approval. Detailed information on the ethical procedures followed by the DHS Program can be found on their official website (http://goo.gl/ny8T6X).

## 3. Results

### 3.1. Prevalence of PNC Utilisation by Mother and Child in SSA

Table 1 reports the prevalence of PNC utilisation by mother and child in SSA in 27 SSA countries. The results show that Malawi has the lowest prevalence of PNC utilisation by mothers (47.48%, 95% CI: 46.60–48.36), whereas Ghana has the highest prevalence of PNC utilisation by mothers (94.88%, 95% CI: 94.18–95.49). Furthermore, Ethiopia has the lowest prevalence of PNC utilisation for the child (43.09%, 95% CI: 41.20–45.01), whereas South Africa has the highest prevalence of PNC utilisation for the child (96.68%, 95% CI: 95.59–97.50).

**Table 1:** Prevalence of PNC utilisation by mother and child in SSA.

	Mother received PNC	Child received PNC
**Country (year of survey)**	**% (95% CI)**	**% (95% CI)**
Angola (2015–2016)	55.53 (53.99–57.05)	57.06 (55.50–58.60)
Burkina Faso (2021)	89.38 (88.57–90.13)	90.29 (89.51–91.01)
Benin (2017–2018)	81.08 (80.17–81.96)	82.60 (81.72–83.46)
Burundi (2016–2017)	58.47 (57.33–59.60)	56.29 (55.14–57.43)
Côte d’Ivoire (2021)	85.60 (84.52–86.63)	84.23 (83.08–85.31)
Cameroon (2018)	78.23 (77.00–79.40)	86.12 (85.08–87.10)
Ethiopia (2016)	49.83 (47.94–51.72)	43.09 (41.20–45.01)
Gabon (2019–2021)	80.39 (78.81–81.88)	91.41 (90.26–92.44)
Ghana (2022)	94.88 (94.18–95.49)	95.55 (94.89–96.13)
Gambia (2019–2020)	94.41 (93.71–95.03)	96.17 (95.57–96.69)
Guinea (2018)	76.53 (74.91–78.08)	79.80 (78.23–81.28)
Liberia (2019–2020)	91.24 (90.24–92.15)	91.27 (90.27–92.18)
Lesotho (2023–2024)	91.87 (90.30–93.21)	93.69 (92.26–94.88)
Madagascar (2021)	90.57 (89.58–91.48)	91.45 (90.49–92.32)
Mali (2018)	74.36 (73.01–75.67)	76.50 (75.17–77.78)
Mauritania (2019–2021)	62.97 (61.66–64.25)	66.61 (65.30–67.90)
Malawi (2015–2016)	47.48 (46.60–48.36)	69.13 (68.31–69.94)
Mozambique (2022–2023)	67.86 (66.27–69.41)	73.26 (71.73–74.74)
Nigeria (2018)	82.91 (82.10–83.68)	82.39 (81.57–83.17)
Rwanda (2019–2020)	73.71 (72.55–74.83)	78.89 (77.81–79.92)
Sierra Leone (2019)	91.83 (91.08–92.53)	92.99 (92.28–93.64)
Senegal (2023)	94.93 (94.25–95.53)	95.20 (94.53–95.79)
Tanzania (2022)	62.82 (61.43–64.19)	66.90 (65.53–68.24)
Uganda (2016)	68.13 (67.07–69.18)	71.71 (70.68–72.72)
South Africa (2016)	90.77 (89.15–92.18)	96.68 (95.59–97.50)
Zambia (2018)	77.90 (76.85–78.92)	83.77 (82.83–84.67)
Zimbabwe (2015)	72.17 (70.76–73.55)	91.61 (90.70–92.44)

### 3.2. Descriptive Summary of the Sample

**Figure 1:**
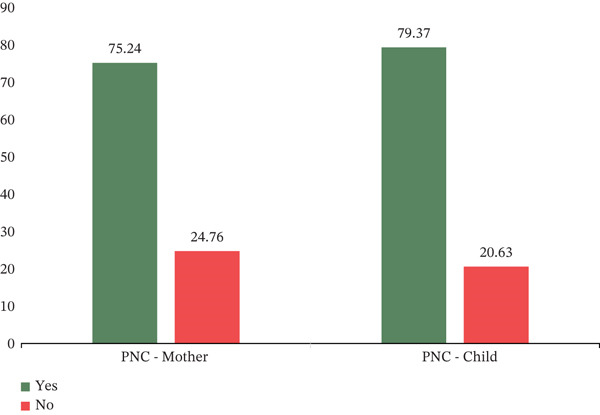
PNC utilisation by mother and child.

Figure 1 reports levels of PNC utilisation by mother and child. It indicates 75.25% of the women utilised PNC. Likewise, 79.37% of the children received PNC.

Table 2 presents the results of the descriptive summary of the sample. It shows that only 8.75% of the women gave birth via caesarean section. In addition, most of them had at least four ANC visits (68.32%). Furthermore, a high proportion of the women have between 1 and 2 children (44.66%), aged between 25 and 29 years (24.86%), have primary education (34.17%), are in the richer wealth category (20.91%), live in rural areas (61.01%), are currently employed (62.46%), reported that distance to health facility is not a problem (65.57%) and do not have health insurance (87.22%).

**Table 2:** Descriptive summary of the sample.

	Total sample	Mother received PNC	Child received PNC
**Variables**	**N (%)**	**N (%)**	**N (%)**
**Caesarean birth**			
No	124,752 (91.25)	91,980 (89.41)	93,476 (90.30)
Yes	11,969 (8.75)	10,892 (10.59)	10,042 (9.70)
**Parity**			
1–2	61,062 (44.66)	46,235 (44.94)	46,894 (45.30)
3–5	53,101 (38.84)	40,079 (38.96)	40,189 (38.82)
≥ 6	22,563 (16.50)	16,563 (16.10)	16,435 (15.88)
**ANC visits**			
< 4 ANC visits	43,320 (31.68)	29,244 (28.43)	29,719 (28.71)
≥ 4 ANC visits	93,406 (68.32)	73,633 (71.57)	73,799 (71.29)
**Age**			
15–19	10,716 (7.84)	7842 (7.62)	8134 (7.86)
20–24	31,893 (23.33)	23,294 (22.64)	24,040 (23.22)
25–29	33,991 (24.86)	25,720 (25.00)	25,683 (24.81)
30–34	27,817 (20.35)	21,238 (20.64)	21,182 (20.46)
35–39	20,227 (14.79)	15,555 (15.12)	15,406 (14.88)
40–44	9243 (6.76)	7063 (6.87)	6982 (6.74)
45–49	2839 (2.08)	2165 (2.10)	2091 (2.02)
**Education level**			
No education	40,465 (29.60)	31,378 (30.50)	30,207 (29.18)
Primary	46,715 (34.17)	31,894 (31.00)	33,163 (32.04)
Secondary	42,876 (31.36)	33,881 (32.93)	34,443 (33.27)
Higher	6670 (4.88)	5724 (5.56)	5705 (5.51)
**Wealth**			
Poorest	25,014 (18.29)	18,404 (17.89)	18,815 (18.18)
Poorer	26,418 (19.32)	19,553 (19.01)	19,908 (19.23)
Middle	28,474 (20.83)	21,125 (20.53)	21,263 (20.54)
Richer	28,589 (20.91)	21,616 (21.01)	21,783 (21.04)
Richest	28,231 (20.65)	22,179 (21.56)	21,749 (21.01)
**Residence**			
Urban	53,310 (38.99)	42,328 (41.14)	41,623 (40.21)
Rural	83,416 (61.01)	60,549 (58.86)	61,895 (59.79)
**Distance to health facility**			
Big problem	47,076 (34.43)	33,739 (32.80)	34,917 (33.73)
Not a big problem	89,650 (65.57)	69,138 (67.20)	68,601 (66.27)
**Wanted pregnancy**			
Then	96,919 (70.89)	74,562 (72.48)	74,128 (71.61)
Later	30,749 (22.49)	22,009 (21.39)	22,904 (22.13)
No more	9058 (6.62)	6306 (6.13)	6486 (6.27)
**Frequency of reading newspaper**			
Not at all	115,790 (84.69)	86,682 (84.26)	86,720 (83.77)
Less than once a week	12,886 (9.42)	10,021 (9.74)	10,429 (10.07)
At least once a week	7736 (5.66)	5933 (5.77)	6155 (5.95)
Almost every day	314 (0.23)	241 (0.23)	214 (0.21)
**Frequency of listening to radio**			
Not at all	59,354 (43.41)	42,663 (41.47)	43,261 (41.79)
Less than once a week	28,934 (21.16)	22,640 (22.01)	22,888 (22.11)
At least once a week	45,448 (33.24)	35,248 (34.26)	35,877 (34.66)
Almost every day	2990 (2.19)	2326 (2.26)	1492 (1.44)
**Frequency of watching television**			
Not at all	74,534 (54.51)	52,725 (51.25)	53,813 (51.98)
Less than once a week	19,333 (14.14)	15,351 (14.92)	15,436 (14.91)
At least once a week	38,530 (28.18)	31,553 (30.67)	31,756 (30.68)
Almost every day	4329 (3.17)	3248 (3.16)	2513 (2.43)
**Currently working**			
No	51,332 (37.54)	37,590 (36.54)	38,350 (37.05)
Yes	85,394 (62.46)	65,287 (63.46)	65,168 (62.95)
**Health insurance**			
No	119,248 (87.22)	88,698 (86.22)	89,145 (86.12)
Yes	17,478 (12.78)	14,179 (13.78)	14,373 (13.88)

Among those who used PNC, the results show that only 10.59% gave birth via caesarean section. Furthermore, the majority of them had at least four ANC visits (71.57%). Also, a high proportion of the women have between 1 and 2 children (44.94%), aged between 25 and 29 years (25%), have secondary education (32.93%), are in the richest wealth category (21.56%), live in rural areas (58.86%), are currently employed (63.46%), reported that distance to the health facility is not a problem (67.20%) and do not have health insurance (86.22%).

### 3.3. Factors Associated With PNC Utilisation for Both Mother and Child in SSA

Table 3 presents results from the multivariable logistic regression analysis on the factors associated with PNC utilisation. Model 1 examines the factors associated with PNC utilisation for mothers, and Model 2 examines the factors associated with PNC utilisation for children.

**Table 3:** Multivariable logistic regression analysis on the factors associated with PNC utilisation for both mother and child in SSA.

	Model 1	Model 2
	**Mother received PNC**	**Child received PNC**
**Variables**	**AOR (95% CI)**	**AOR (95% CI)**
**Caesarean birth**		
No	Ref	Ref
Yes	3.64 (3.33–3.98) ^∗∗∗^	2.41 (2.21–2.64) ^∗∗∗^
**Parity**		
1–2	Ref	Ref
3–5	1.02 (0.97–1.06)	0.94 (0.90–0.99) ^∗^
≥ 6	0.98 (0.91–1.05)	0.92 (0.85–0.99) ^∗^
**ANC visits**		
< 4 ANC visits	Ref	Ref
≥ 4 ANC visits	1.24 (1.20–1.29) ^∗∗∗^	1.27 (1.22–1.32) ^∗∗∗^
**Age**		
15–19	Ref	Ref
20–24	0.97 (0.91–1.04)	0.98 (0.91–1.05)
25–29	1.01 (0.94–1.09)	0.98 (0.90–1.07)
30–34	1.06 (0.98–1.16)	1.05 (0.96–1.16)
35–39	1.08 (0.98–1.18)	1.04 (0.94–1.15)
40–44	1.13 (1.01–1.25) ^∗^	1.09 (0.97–1.22)
45–49	1.15 (1.00–1.32) ^∗^	1.09 (0.94–1.27)
**Education level**		
No education	Ref	Ref
Primary	1.02 (0.97–1.07)	1.02 (0.97–1.07)
Secondary	1.12 (1.05–.18) ^∗∗∗^	1.09 (1.03–1.16) ^∗∗^
Higher	1.32 (1.17–1.49) ^∗∗∗^	1.24 (1.09–1.40) ^∗∗∗^
**Wealth**		
Poorest	Ref	Ref
Poorer	1.02 (0.96–1.08)	1.01 (0.95–1.07)
Middle	1.00 (0.94–1.06)	0.98 (0.92–1.05)
Richer	1.02 (0.96–1.10)	1.00 (0.93–1.07)
Richest	1.14 (1.04–1.24) ^∗∗^	1.09 (0.99–1.19)
**Residence**		
Urban	Ref	Ref
Rural	0.95 (0.88–1.01)	0.97 (0.90–1.05)
**Distance to health facility**		
Big problem	Ref	Ref
Not a big problem	1.07 (1.02–1.12) ^∗∗^	1.02 (0.97–1.07)
**Wanted pregnancy**		
Then	Ref	Ref
Later	0.91 (0.87–0.95) ^∗∗∗^	0.97 (0.93–1.02)
No more	0.85 (0.79–0.91) ^∗∗∗^	0.84 (0.78–0.91) ^∗∗∗^
**Frequency of reading newspaper**		
Not at all	Ref	Ref
Less than once a week	1.09 (1.02–1.17) ^∗∗^	1.13 (1.05–1.22) ^∗∗^
At least once a week	1.11 (1.02–1.21) ^∗^	1.16 (1.05–1.27) ^∗∗^
Almost every day	1.54 (1.02–2.33) ^∗^	1.40 (0.92–2.13)
**Frequency of listening to radio**		
Not at all	Ref	Ref
Less than once a week	1.12 (1.06–1.18) ^∗∗∗^	1.20 (1.14–1.27) ^∗∗∗^
At least once a week	1.15 (1.10–1.21) ^∗∗∗^	1.26 (1.19–1.32) ^∗∗∗^
Almost every day	1.12 (0.94–1.33)	1.46 (1.17–1.82) ^∗∗∗^
**Frequency of watching television**		
Not at all	Ref	Ref
Less than once a week	1.09 (1.03–1.16) ^∗∗^	1.11 (1.04–1.19) ^∗∗^
At least once a week	1.09 (1.03–1.16) ^∗∗^	1.15 (1.08–1.24) ^∗∗∗^
Almost every day	1.45 (1.24–1.70) ^∗∗∗^	1.83 (1.50–2.23) ^∗∗∗^
**Currently working**		
No	Ref	Ref
Yes	1.22 (1.17–1.27) ^∗∗∗^	1.27 (1.22–1.33) ^∗∗∗^
**Health insurance**		
No	Ref	Ref
Yes	1.15 (1.06–1.25) ^∗∗∗^	1.10 (1.01–1.19) ^∗^
**Constant**	0.60 (0.51–0.71) ^∗∗∗^	0.58 (0.48–0.69) ^∗∗∗^

*Note: N* **=** 136,721. 95% confidence interval (CI) in parentheses.

Abbreviations: AOR, adjusted odds ratio; Ref, reference group.

^∗^p < 0.05.

^∗∗^p < 0.01.

^∗∗∗^p < 0.001.

The results indicate that women who delivered via caesarean section are over three times more likely to receive PNC compared with those who did not (AOR = 3.64; 95% CI: 3.33–3.98). Similarly, their children are significantly more likely to receive PNC (AOR = 2.41; 95% CI: 2.21–2.64).

Women who had four or more ANC visits are more likely to receive PNC (AOR = 1.24; 95% CI: 1.20–1.29), and their children also have greater odds of receiving PNC (AOR = 1.27; 95% CI: 1.22–1.32) than those who had less than four ANC visits.

Women with higher education are more likely to receive PNC for themselves (AOR = 1.32; 95% CI: 1.17–1.49) and their children (AOR = 1.24; 95% CI: 1.09–1.40) compared with those with no education.

Women who reported that they did not want the pregnancy are less likely to receive PNC for themselves (AOR = 0.85; 95% CI: 0.79–0.91) and their children (AOR = 0.84; 95% CI: 0.78–0.91) than those who wanted it.

Exposure to media also emerged as a significant factor of PNC utilisation. Women who read newspapers at least once a week are more likely to receive PNC for themselves (AOR = 1.11; 95% CI: 1.02–1.21) and their children (AOR = 1.16; 95% CI: 1.05–1.27) compared with those who do not read newspapers. Similarly, women who listen to the radio at least once a week are more likely to receive PNC for themselves (AOR = 1.15; 95% CI: 1.10–1.21) and their children (AOR = 1.26; 95% CI: 1.19–1.32) compared with those who do not listen to the radio. In addition, women who watch television almost every day are more likely to receive PNC for themselves (AOR = 1.45; 95% CI: 1.24–1.70) and for their children (AOR = 1.83; 95% CI: 1.50–2.23) compared with those who do not.

Currently working women have significantly higher odds of accessing PNC for themselves (AOR = 1.22; 95% CI: 1.17–1.27) and their children (AOR = 1.27; 95% CI: 1.22–1.33) compared with the unemployed. Similarly, women with health insurance coverage are more likely to receive PNC for themselves (AOR = 1.15; 95% CI: 1.06–1.25) and their children (AOR = 1.10; 95% CI: 1.01–1.19) compared with those with no health insurance.

## 4. Discussion

This study sought to investigate the factors associated with PNC utilisation for both mothers and their newborns in SSA. The findings revealed that a range of clinical, sociodemographic, and access‐related factors influence whether women and their children receive PNC services. The implications are important for policy reforms aiming to improve maternal and newborn survival in the region.

One of the strongest factors associated with PNC utilisation identified in the study is caesarean section delivery, which significantly increases the likelihood of receiving PNC for both mother and child. This finding is consistent with earlier studies in Zambia and Ethiopia [1, 20, 27], which observed that women who undergo caesarean deliveries often have increased medical follow‐ups due to postsurgical care requirements. Health systems tend to schedule routine checks to monitor recovery, thus increasing PNC uptake. Moreover, caesarean delivery is more common among urban and wealthier women who tend to have better access to healthcare services, further explaining the association. However, this also indicates a disparity, where those who deliver vaginally often in rural or low‐resource settings may be overlooked, underscoring the need for proactive outreach to this group.

ANC attendance also emerged as a key factor. Women who had four or more ANC visits were significantly more likely to utilise PNC services. In this study, the ≥ 4 visits threshold reflects the measure available in the DHS dataset and is widely used as a proxy for adequate ANC utilisation in similar analyses [31, 32], even though the WHO′s 2016 guidelines recommend a minimum of eight ANC contacts. Several studies have demonstrated that frequent ANC attendance enhances awareness of maternal and newborn health services, fosters trust in the healthcare system and subsequently increases the likelihood of PNC utilisation [31–33]. ANC offers a platform to counsel women on birth preparedness and the importance of postnatal follow‐up, thus enhancing service continuity. The current finding reinforces the value of ANC as a critical entry point into the maternal healthcare system.

In terms of maternal education, the analysis confirmed that higher education levels significantly increase the likelihood of mothers and their children receiving PNC. This aligns with extensive empirical evidence across sub‐Saharan Africa. For instance, Wang et al. [34] found that women with secondary or higher education in the Democratic Republic of the Congo were more likely to utilise both antenatal and PNC services. Similarly, Tiruneh et al. [35] reported that in Ethiopia, educated women had significantly greater odds of using PNC services. A broader West African analysis by Habte et al. [36] further confirmed that maternal education remains a consistent predictor of maternal service uptake across diverse contexts. These findings reinforce the conclusion that education enhances women′s knowledge, decision‐making capacity and access to health information, all of which contribute to increased utilisation of PNC services.

Contrastingly, the study found that unwanted pregnancies were significantly associated with reduced PNC utilisation. This is supported by research from Tanzania and Mozambique, which suggests that women with unintended pregnancies are less motivated to seek healthcare services due to emotional stress, stigma or lack of social support [37–39]. These women may also delay seeking ANC and are more likely to deliver at home, thereby missing key touchpoints for PNC. This finding highlights the need for improved access to family planning services and psychosocial support, especially during and after unintended pregnancies.

Another crucial factor identified is media exposure. Women who regularly read newspapers, listen to the radio or watch television were more likely to receive PNC services for themselves and their children. This reinforces the evidence that mass media plays an influential role in health education and behaviour change [31, 40, 41]. Media platforms often serve as key sources of information on maternal and child health, promoting awareness about the availability and importance of services such as PNC [41]. Notably, television was the most impactful medium, possibly due to its visual appeal and ability to communicate complex information more effectively. This presents an opportunity for governments and partners to scale up targeted media campaigns to improve service uptake, especially in rural areas. Although our findings show that women exposed to media were more likely to utilise PNC services, this association should indeed be interpreted with caution. Media exposure may act as a proxy for broader socioeconomic advantages rather than representing a direct causal pathway from health information to behaviour change.

Employment status was a significant factor of PNC utilisation, with currently working women showing higher odds of accessing PNC services for themselves and their children. This aligns with findings from Somefun and Ibisomi [11] and Tessema et al. [27], which indicate that employment enhances a woman′s financial autonomy, improves her health‐seeking behaviour and increases her decision‐making power within the household. Furthermore, Shen et al. [42] emphasize that employed women are more likely to be exposed to health‐related information through social networks, workplaces and mass media. Igyuse et al. [43] also highlight that employment status positively influences maternal health services utilisation, particularly among women in urban and semiurban settings. However, this trend also points to a gap in access, where economically disadvantaged or unemployed women face logistical and financial constraints that hinder their ability to seek care, particularly in contexts lacking universal health coverage or where indirect costs such as transport remain a barrier. Therefore, policy efforts must aim to bridge this equity gap by subsidizing PNC services and implementing outreach strategies targeting nonworking and rural populations.

Health insurance coverage was positively associated with PNC utilisation, supporting existing evidence from countries such as Ghana, Nigeria and Rwanda, where national health insurance schemes have lowered financial barriers to maternal healthcare [44–46]. Insurance facilitates access by reducing out‐of‐pocket payments and improving the affordability of skilled care [41]. In Ghana, for instance, insured women are more likely to deliver in health facilities and seek postnatal services compared with uninsured women [45]. However, some studies caution that insurance alone is not always sufficient. Khatiwada et al. [47] found that even insured women may delay or forego PNC if facilities are understaffed or geographically inaccessible. Additionally, coverage disparities between urban and rural populations or between formal and informal workers persist. These gaps point to the need for not only expanding coverage but also ensuring service quality and provider availability, especially in underserved areas.

Regarding parity, the study observed that women with three or more children were slightly less likely to seek PNC for their youngest child. This is consistent with Olorunsaiye et al. [48] and Ononokpono and Odimegwu [49], who noted that multiparous women often perceive less need for follow‐up care due to prior experience with childbirth and child‐rearing. However, contrasting evidence from Sacks et al. (2016) shows that higher‐parity women may be more likely to seek PNC owing to cumulative awareness gained through repeated interactions with the health system. These conflicting findings likely reflect contextual differences, including cultural norms, household decision‐making dynamics and variations in the availability and quality of health services. Taken together, the evidence suggests that the relationship between parity and PNC utilisation is highly context‐specific and highlights the need for tailored communication strategies that address the distinct needs of both first time and experienced mothers.

In terms of maternal age, older women (40–49 years) showed a slightly higher likelihood of utilising PNC, possibly due to greater risk awareness, health literacy and decision‐making autonomy, as supported by findings from Ghana, South Sudan and Indonesia [41, 50, 51]. However, other studies, such as Kebede et al. [52] in Ethiopia, found that younger mothers were more likely to seek PNC, often due to anxiety related to first‐time motherhood and stronger support systems. Additionally, Dhakal et al. [53] and Workineh et al. [54] observed that older women may underutilise PNC due to negative past experiences or cultural beliefs that perceive pregnancy as a natural state not requiring medical attention. These mixed findings suggest that the influence of maternal age on PNC utilisation is not uniform but shaped by broader social and cultural contexts, underscoring the need for age‐sensitive approaches that respond to the specific barriers and motivations experienced by different age groups.

### 4.1. Strengths and Limitations of the Study

This study is timely as it reveals key critical factors that predict PNC service utilisation in SSA, a poor‐resource setting. In the light of scarce resources competing for several demands, the results could benefit health ministries within SSA to restrategise on tailor‐made interventions targeted at PNC uptake to benefit mothers and their newborns. The study employed appropriate statistical methods in the investigation and reporting standards, which improve the robustness of the findings and conclusions. However, the results should be interpreted with caution, especially as the study relied on datasets generated from a cross‐sectional survey; causality cannot be established. In addition, social desirability bias cannot be overlooked. Moreover, about two‐thirds of respondents (66%) were excluded due to missing data. This level of missingness may have introduced selection bias, as the analytic sample may not fully represent all subgroups of women. As a result, estimates of PNC utilisation may be either overestimated or underestimated. Furthermore, the DHS measure used in this analysis captures only immediate PNC checks received before discharge from the health facility. Consequently, the study does not account for subsequent PNC contacts that occur within the recommended 6‐week postpartum period. As a result, the measured prevalence may overestimate true PNC utilisation across the entire continuum of care. Additionally, the DHS data used in this analysis do not include detailed health system or facility‐level characteristics such as staffing levels, patient load or service readiness, which may influence whether mothers and newborns receive a postnatal health check before discharge. Therefore, the analysis was limited to individual and household‐level factors. Future studies incorporating health facility and health system data would provide a more comprehensive understanding of determinants of immediate PNC provision.

## 5. Conclusion

PNC utilisation in SSA is influenced by a combination of factors related to healthcare access, maternal characteristics, socioeconomic status and information exposure. Caesarean birth, ANC attendance, education, employment, media exposure and insurance coverage are all key facilitators of PNC use, whereas high parity and unintended pregnancies reduce uptake. The limited influence of wealth and urban–rural residence in the adjusted model suggests that more direct enablers, such as education and health system contact, are increasingly important. These findings highlight several entry points for interventions, including strengthening ANC and PNC counselling, expanding insurance coverage, improving female education and leveraging mass media for maternal health promotion. A stronger emphasis on reaching vulnerable groups, especially women with unintended pregnancies, lower parity and those outside the formal economy, is essential for improving maternal and newborn health outcomes across SSA.

## Author Contributions

S.Y. contributed to the study design. A.B. performed the analyses. S.Y., M.S., L.B. and F.A. jointly drafted the literature review, methods, results and discussion sections of the manuscript.

## Funding

No funding was received for this manuscript.

## Disclosure

S.Y. had the final responsibility to submit for publication. All authors read and approved the final manuscript.

## Ethics Statement

Ethics approval for this study was not required since the data is secondary and is available in the public domain. More details regarding DHS data and ethical standards are available at: http://goo.gl/ny8T6X.

## Consent

No consent to publish was needed for this study, as we did not use any details, images or videos related to individual participants. In addition, the data used is available in the public domain.

## Conflicts of Interest

The authors declare no conflicts of interest.

## Supporting information


**Supporting Information** Additional supporting information can be found online in the Supporting Information section. Table S1 Characteristics of the excluded sample.

## Data Availability

Data for this study were sourced from DHS and are available here: http://dhsprogram.com/data/available-datasets.cfm.
