# Supplementary material for: Left Behind After Birth: A Cross‐Sectional Analysis of Factors Associated With Postnatal Care Use in Sub‐Saharan Africa
Source: J Pregnancy. 2026 Apr 24;2026:3035162. doi: 10.1155/jp/3035162 (PMC13109620; doi:10.1155/jp/3035162)
Supplement: Supplementary file 1 — Supporting Information Additional supporting information can be found online in the Supporting Information section. Table S1 Characteristics of the excluded sample. [file JP-2026-3035162-s001.docx]

**Table S1: Characteristics of excluded sample**

| **Variables** | **N** | **%** |
| --- | --- | --- |
| **Caesarean birth** |  |  |
| No | 54,503 | 100.00 |
| **Parity** |  |  |
| No Child | 115,585 | 42.52 |
| 1 - 2 | 50,683 | 18.64 |
| 3 - 5 | 63,558 | 23.38 |
| ≥6 | 42,042 | 15.46 |
| **ANC visits** |  |  |
| <4 ANC visits | 33,331 | 61.48 |
| ≥4 ANC visits | 20,882 | 38.52 |
| **Age** |  |  |
| 15-19 | 78,321 | 29.01 |
| 20-24 | 42,236 | 15.64 |
| 25-29 | 32,973 | 12.21 |
| 30-34 | 28,945 | 10.72 |
| 35-39 | 29,995 | 11.11 |
| 40-44 | 29,138 | 10.79 |
| 45-49 | 28,414 | 10.52 |
| **Education level** |  |  |
| No education | 84,163 | 30.96 |
| Primary | 79,013 | 29.06 |
| Secondary | 94,756 | 34.85 |
| Higher | 13,936 | 5.13 |
| **Wealth** |  |  |
| Poorest | 55,855 | 20.54 |
| Poorer | 51,062 | 18.78 |
| Middle | 52,356 | 19.26 |
| Richer | 52,186 | 19.20 |
| Richest | 60,409 | 22.22 |
| **Residence** |  |  |
| Urban | 109,020 | 40.10 |
| Rural | 162,848 | 59.90 |
| **Distance to health facility** |  |  |
| Big problem | 99,325 | 37.29 |
| Not a big problem | 167,058 | 62.71 |
| **Wanted pregnancy** |  |  |
| Then | 44,419 | 76.87 |
| Later | 9,715 | 16.81 |
| No more | 3,649 | 6.32 |
| **Frequency of reading newspaper** |  |  |
| Not at all | 226,408 | 83.28 |
| Less than once a week | 27,236 | 10.02 |
| At least once a week | 17,659 | 6.50 |
| Almost every day | 560 | 0.21 |
| **Frequency of listening to radio** |  |  |
| Not at all | 129,473 | 47.62 |
| Less than once a week | 57,549 | 21.17 |
| At least once a week | 81,791 | 30.08 |
| Almost every day | 3,055 | 1.12 |
| **Frequency of watching television** |  |  |
| Not at all | 145,481 | 53.51 |
| Less than once a week | 39,114 | 14.39 |
| At least once a week | 82,052 | 30.18 |
| Almost every day | 5,221 | 1.92 |
| **Currently working** |  |  |
| No | 121,582 | 44.89 |
| Yes | 149,250 | 55.11 |
| **Health insurance** |  |  |
| No | 235,455 | 88.39 |
| Yes | 30,928 | 11.61 |
